# Delivering integrated diabetes and mental healthcare for people with type 1 diabetes disordered eating (T1DE): a mixed methods evaluation

**DOI:** 10.1136/bmjopen-2025-107381

**Published:** 2026-03-09

**Authors:** Elaine F Harkness, Saima Bashir, Maartje Kletter, Stephanie Gillibrand, Paul Wilson, Jo Dumville, Peter Bower

**Affiliations:** 1 NIHR Rapid Service Evaluation Team, School of Health Sciences, Faculty of Biology, Medicine and Health, The University of Manchester, Manchester, UK; 2 School of Health Sciences, Faculty of Biology, Medicine and Health, The University of Manchester, Manchester, UK

**Keywords:** DIABETES & ENDOCRINOLOGY, MENTAL HEALTH, Eating disorders, Delivery of Health Care, Integrated, Implementation Science

## Abstract

**Abstract:**

**Objectives:**

People with type 1 diabetes may be at increased risk of disordered eating, which may increase risk of elevated poor outcomes and high-risk complications. Type 1 diabetes disordered eating (T1DE) services were set-up to integrate diabetes and mental healthcare to better support people with T1DE and improve longer-term outcomes. A rapid evaluation was conducted to explore the implementation of T1DE services. Specifically, we aimed to: describe service delivery models; investigate staff experiences of impact and delivery of implementation; explore patient experiences of T1DE services; and to report health outcomes and associated costs.

**Design:**

Rapid evaluation using mixed methods (service mapping, staff and patient interviews, staff survey, analysis of clinical and economic data). Health outcome data was reported at baseline and 6 months.

**Setting:**

This study explored the implementation of five new T1DE services and three existing services.

**Participants:**

Staff working within T1DE services and patients who received care from T1DE services.

**Results:**

Assessment of our mixed methods study identified four key findings: (1) T1DE delivery models: The T1DE services displayed modest variation in models of delivery, but similarities were more evident, with a focus on direct delivery to patients involving joint meetings between diabetes and mental health staff. Nevertheless, some services also took on a ‘consultation’ role, providing advice and support to wider staff outside the service also managing these patients. Delayed implementation of the services slowed the formation of fully integrated teams and the ability of services to operate at scale. (2) Staff experience: Workforce issues were a crucial aspect of T1DE pilots. Managing this patient population is associated with high levels of anxiety for staff. Nevertheless, once formed, staff reported a very positive experience of working in integrated teams. (3) Patient experience: Although only a small sample of patients were interviewed, they reported a profoundly different experience to their previous care, which was now perceived as supportive and relationship focused. Although such improvements were aligned with the integrated care model underlying T1DE, it was less clear how such changes in patient experience would feature in decisions about commissioning. (4) Health outcomes and associated costs: There were 139 patients accepted onto the care pathway. Improvements were seen for all health outcomes. Compared with baseline measures, there was a mean 0.97% reduction in HbA1c (glycated haemoglobin) from 11.2% to 10.2% at 6 months. Improvements were also seen in other outcomes, including the diabetes eating problem scale and the diabetes distress scale. However, the number of patients on the care pathway with follow-up at 6 months was relatively small (n=29–47) and definitive statements about clinical or cost-effectiveness were not possible.

**Conclusions:**

Overall, T1DE services were well received by both staff and patients. Due to a number of logistical challenges, the implementation of services was slower than anticipated, resulting in a limited number of patients on the care pathway. Securing local funding for existing services, once national pilot funding ended, was identified as a significant challenge. In order to ensure services are sustainable and commissioned at a local level, consideration may need to be given to alternative service delivery models.

**STRENGTHS AND LIMITATIONS OF THIS STUDY**

- The implementation of integrated physical and mental healthcare for people with type 1 diabetes disordered eating (T1DE) was explored in a number of services from the perspective of both staff and patients.
- The study recruited a large sample of staff from T1DE services.
- Interviews were conducted at an early stage of implementation and therefore views and experiences may change over time.
- Patient interviews were conducted primarily from one service.

## Introduction

 Diabetes is one of the most common chronic conditions in the UK with approximately 8% of cases having type 1 diabetes.^1^ Type 1 diabetes commonly manifests during adolescence. At any one time, around 400 000 people in the UK are estimated to have type 1 diabetes. Diagnosis means initiation of insulin therapy and modification of eating habits to optimise glycaemic control. Adolescence is also the most common period for the onset of eating disorders. A meta-analysis of 94 studies estimated the lifetime prevalence of an eating disorder as 8.4% (95% CI 3.3% to 18.6%) for women and 2.2% (95% CI 0.8% to 6.5%) for men.^2^

The increased focus on eating patterns and food intake by people with type 1 diabetes, alongside the need for daily insulin and negative experiences from their diagnosis, may impact on eating practices and related behaviours. People with type 1 diabetes may be at increased risk of disordered eating, known as type 1 diabetes disordered eating (T1DE). A meta-analysis of 14 studies showed that the relative risk (RR) of an eating disorder was 2.47 (95% CI 1.84 to 3.32) in people with type 1 diabetes compared with non-diabetic controls, with a higher risk for bulimia nervosa and binge eating.^3^

Insulin restriction or omission to promote weight loss is also a recognised issue in people with T1DE: sometimes called diabulima.^4^ The prevalence of insulin omission/misuse is approximately 10.3% (95% CI 8.3% to 13.0%) with this behaviour being much more prevalent in females (RR=14.21, 95% CI 2.66 to 76.04 for insulin omission; RR=6.51, 95% CI 1.14 to 37.31 for insulin misuse).^3^ Induction of hyperglycaemia, for example, through insulin omission means glucose is excreted rather than absorbed, which can lead to weight loss but also serious health problems. These include increased risk of mortality, diabetes ketoacidosis (DKA) and premature onset of complications of diabetes such as retinopathy and nephropathy.

Systematic reviews have shown a lack of evidence-based approaches to support people with T1DE.^5^ With growing recognition of unmet need and lack of evidence on how to manage this complex patient group, National Health Service (NHS) England commissioned a pilot of two T1DE services in 2019. The key principle of the T1DE pilots was to provide integrated physical and mental healthcare in an effort to provide effective treatment and care for this patient group. The evaluation of these services was conducted during the COVID-19 pandemic and reported data on 69 patients accepted onto the pathway^6^ leading to challenges in fully understanding the impact of services. Despite this, there were statistically significant reductions in glycated haemoglobin (HbA1c) of 2.3% (range: reduction of 7.1% to increase of 0.4%, n=11) and 2.5% (range: reduction of 8.9% to increase of 2.4%, n=14) in the two services after 12 months of follow-up. There were also improvements in other health outcomes (eg, diabetes disordered eating, depression and anxiety), but samples were small and demonstrated wide variability. Patients and staff were generally supportive of the services delivered.

The National Institute for Health and Care Research-funded Rapid Service Evaluation Team at the University of Manchester was commissioned to conduct a further evaluation of T1DE services. We aimed to: describe service delivery models (across services); investigate staff experiences of impact and delivery of implementation; explore patient experiences of T1DE services; and to report health outcomes and associated costs (across services).

## Methods

### Study design

The current evaluation used mixed methods to explore the early (five new services) and ongoing (three existing services) implementation of T1DE services. We conducted interviews with staff working in T1DE services and patients receiving care. We also conducted a survey to gather staff views on service implementation. Clinical data from new T1DE services was supplied by analysts at NHS England using a bespoke minimum dataset (MDS).

To inform our evaluation design, we met with stakeholders, including the NHS England Diabetes team.

To meet our objectives, the following methods were used:

#### T1DE delivery models

We met informally with staff members from individual T1DE services to gather information on wider contextual factors surrounding implementation of the T1DE services. Details of staff employed by the services were collated using the health economic questionnaires as detailed under the health economic analysis below.

#### Staff experience

Views and experiences of staff were conducted using qualitative interviews and surveys.

Interviews were conducted with staff members from new and existing T1DE services between September 2023 and July 2024. The topic guide (online supplemental appendix 1) was informed by core constructs from the Consolidated Framework for Implementation Research applying Damschroder *et al*^7^ and the Health Disparities framework.^8^ Staff interviews explored:

- Staff views towards and acceptability of T1DE.
- Barriers and facilitators to implementing the service.
- The impact of the T1DE service on staff and patients.
- Anticipated outcomes and benefits for patients.
- Unintended consequences of the implementation of the T1DE service.

The staff survey included the validated Normalisation MeAsure Development questionnaire (NoMAD).^9^ NoMAD was designed to measure constructs of Normalisation Process Theory (NPT), which explores factors that support or inhibit embedding of new practices into normal care.^10^ NPT constructs include: coherence (how do individuals and groups make sense of new practices); cognitive participation (do participants see new practices as legitimate); collective action (what work do participants do to enact new practices); and reflexive monitoring (how do participants make sense of the effects of new practices). Survey participants were also asked to complete the Acceptability of Intervention Measure, Intervention Appropriateness Measure and Feasibility of Intervention Measure (FIM)^11^ (online supplemental appendix 2).

Staff were asked to complete the survey in February and November 2024. The follow-up survey was only conducted for staff in new T1DE services due to the closure of two of the three existing services.

#### Patient experience

Interviews were conducted with patients in receipt of care from the T1DE services. Patients were approached by their local T1DE services to invite them to take part in the study.

Interviews were informed by a semi-structured topic guide (online supplemental appendix 3) which was developed in conjunction with the Patient and Public Involvement and Engagement (PPIE) advisory group (see PPIE statement) and explored:

- Experiences of previous care and expectations from the T1DE service.
- Care received and perception of integration of the T1DE service.
- Important outcomes from the patient perspective.
- Discharge and discharge planning.

Participants were also asked to complete demographic information at the time of interview.

For both staff and patient interviews, informed (verbal) consent was obtained from all participants. All interviews were conducted remotely (online via Microsoft Teams or telephone call) and were audio-recorded using an encrypted handheld device. Interviews were professionally transcribed by a University of Manchester approved supplier.

#### Health outcomes and associated costs

##### NHS England Minimum DataSet

NHS England developed a T1DE specific MDS to collect patient-level data at baseline and at follow-up. The dataset contained demographic information including age and ethnicity. Clinical measures included:

- HbA1c, a measure of average blood glucose (sugar) levels: an ideal HbA1c is below 48 mmol/mol (6.5%). A change of 5.5 mmol/mol (equivalent to 0.5%) is typically considered as clinically significant.^12 13^
- Body mass index (BMI) calculated from reported height and weight where a BMI between 18.5 and <25 kg/m^2^ is considered within the healthy range.
- Diabetes Eating Problem Scale – Revised (DEPS-R)^14^ is a 16-item self-reported questionnaire designed to screen for disordered eating behaviours, scored between 0 and 80, a score of 20 or more is used to identify patients at risk of T1DE.
- Diabetes Distress Scale – 2 (DDS-2)^15^ is a 2-item scale used to rate concerns about the emotional burden of diabetes, scored between 1 and 6, with a score of 3 or more indicating high diabetes distress.
- Generalised Anxiety Disorder (GAD7) questionnaire^16^ is a 7-item screening tool, scored between 0 and 21, with a score of 10 or more indicating generalised anxiety disorder. A change of 4 points or more is considered clinically significant.^17^
- Patient Health Questionnaire (PHQ9)^18^ assessed on a 9-item scale, scored between 0 to 27, with a score of 15 or more indicating moderate or severe depression. A change of 6 points or more is considered clinically significant.^17^
- EQ-5D-5L measures health-related quality of life across five dimensions (mobility, self-care, usual activities, pain/discomfort, anxiety/depression) each with five levels of severity.^19^ This score is converted into a single country-specific EQ-5D index score (utility value) ranging from below 0 (worse than dead) to 1.0 (full health).
- Work and Social Adjustment Scale (WSAS)^20^ is a 5-item scale, scored between 0 and 40, with scores over 10 indicating significant functional impairment.

We worked with NHS England analysts who conducted a descriptive analysis from the MDS.

##### Health economic analysis

A cost-consequence analysis was used to explore service resource use and associated cost data alongside interim changes in health outcomes using anonymised aggregated clinical data from the MDS (as detailed above) and cost (pay and non-pay) data collected from all sites. Cost consequence analysis involves clear presentation of how the services impact various measures (which could include activity and cost implications alongside a range of outcomes) but without formal integration of these data. That is, no single measure is generated, unlike the output of a cost-effectiveness analysis. The cost consequence analysis primarily focuses on NHS costs associated with the implementation of the service and the improvements in health outcomes. The cost perspective is confined to the health system, excluding capital costs and patient-related direct and indirect costs (eg, working days lost).

To collect information on staff input and other healthcare resources (pay and non-pay costs), a detailed questionnaire was distributed to key contacts in each service (see online supplemental appendix 4). For staff pay costs, roles included: consultant diabetologist, diabetes specialist nurse, diabetes dietitian, psychiatrist, psychotherapist, psychologist, eating disorder dietitian, eating disorder therapist, mental health nurse, medical consultant, project manager and administrative staff. These are expressed in terms of % full-time equivalent and grade.

For non-pay costs, the questionnaire included items such as venue expenses, travel costs, information technology equipment, evaluation and analysis, staff training (T1DE team), engagement activities, outreach and educational materials (external to the T1DE team), phones and mobile devices, consumables, communications about the service (including website setup and referral pathways), overheads and any additional unbudgeted items. Capital costs are excluded from this analysis.

### Analysis

We rapidly analysed data from staff and patient interviews. Coding was conducted using a modified version of the framework analysis.^21^ We identified themes and subthemes inductively, based on similarity^21 22^ where themes were inductively deduced to generate a set of themes and subthemes. The themes and sub-themes were subsequently added to a matrix (framework).

Data relating to staffing levels, staff survey and patient health-related outcomes were reported descriptively. No significance testing or 95% CIs were calculated.

### PPIE statement

We formed an advisory panel consisting of four individuals with lived experience of T1DE identified via the Juvenile Diabetes Research Foundation (JDRF - now known as Breakthrough T1D: Type 1 Diabetes). Three healthcare professionals also agreed to participate (also identified via JDRF). The advisory panel was consulted at a number of time points during the evaluation, providing useful context around T1DE and input into participant information leaflets, distress protocols and staff and service user topic guides.

Details of the project were also presented to the Greater Manchester Applied Research Collaborative young persons advisory group at the start of the project. The group provided input to staff and service user topic guides.

### Positionality statement

None of the research team had direct lived experience of T1DE or previous research experience in this specific topic area. Throughout the research process, we engaged with our PPIE advisory group which was made up of people (male and female) with lived experience of T1DE, as well as healthcare professionals working in T1DE. We also worked closely with NHS stakeholders. This helped to give context to the research team and to ensure we addressed the right questions during the research process. The research team also met regularly during the data collection and analysis to discuss findings and reflect as a group.

## Results

We present the mixed methods data under four headings: (1) description of the T1DE delivery models in each site and their similarities and differences, (2) staff experience, (3) patient experience and (4) health outcomes and associated costs.

### T1DE delivery models

Two new T1DE services were based in eating disorder clinics, two in diabetes clinics and one in a mental health clinic with an onsite eating disorders unit. Clinical leads were specialists in the type of setting where services were based, although in two services there were joint clinical leads with expertise in mental health or eating disorders and diabetes. One new service had a small pre-existing, but well-established T1DE service prior to receipt of NHS England pilot funding with a consultant in eating disorders as the clinical lead.

Several services initially struggled to recruit key staff (particularly psychologists). Other issues relating to staffing of services included the part-time nature of many of the roles, junior doctor strikes, recruitment freezes and job insecurity (short-term contracts). Two services which had originally planned to operate at more than one site were scaled back due to limited capacity and one service struggled to get local eating disorder input. Services also reported additional challenges in terms of working across organisational boundaries (eg, mental health and acute Trusts) and the issues this posed in terms of line management arrangements.

These workforce issues slowed the formation of fully integrated teams and the ability of services to operate at scale. While two services opted to provide some form of service at an early stage, despite not having a full complement of staff, this meant the level of therapy/treatment was restricted but some form of support was available to patients if required.

Table 1 shows the staff employed and full-time equivalents for each of the new T1DE services at the time of the evaluation. All services employed a consultant diabetologist, diabetes specialist nurse and dietitian (specialist diabetes or eating disorder dietitian). All but one service had access to a psychologist, which instead employed a full-time eating disorder therapist. Three services had input from a psychiatrist and two from a mental health nurse. Most services also had administrative or project management support. Total full-time equivalents ranged from 2.1 to 4.2.

**Table 1:** Staff employed and FTEs by T1DE services

Staffing (FTE)	Service				
	1	2	3	4	5
Diabetes	0.9	1.6	1.6	1.0	1.1
Mental health	0.6	0.6	1.0	0.75	0.5
Eating disorders	0	0	0.08	1.8	1.6
Admin/other	0.6	1.0	0.9	0.0	1.0
Total	2.1	3.2	3.6	3.6	4.2

FTEs, full-time equivalents; T1DE, type 1 diabetes disordered eating.

T1DE delivery models displayed modest variation, with similarities being more evident with a focus on direct delivery to patients with joint meetings between diabetes and mental health staff. However, some services also took on a ‘consultation’ role, providing advice and support to wider staff outside the services who were also involved in managing these patients. Existing services talked about how they had been involved in the development of new guidelines (eg, Medical Emergencies in Eating Disorders - MEED) and developing a specific training package for T1DE. Staff at some services talked about delivering short training sessions to other team members and to wider networks.

While most services initially focused on referrals from diabetes and eating disorder services where T1DE services were located, one service planned to use an outreach model based within primary care, meeting with general practices (and community diabetes specialist nurses under primary care but recruited through Trusts) to screen lists of diabetes patients to identify potential eligible referrals.

Integration of care within services took the form of MDT meetings, held weekly or 2 weekly to discuss individual patients, joint clinic appointments (eg, diabetes specialist nurse and dietitian or diabetes specialist nurse and psychiatrist) with patients, peer supervision and other mechanisms for building cohesive relationships within teams (eg, one service had a set day when all team members were in).

### Staff experience

Interviews were conducted with 18 members of staff from new T1DE services and 7 from existing services (average duration 56 min). Interviewees included consultant psychiatrists, consultant diabetologists, clinical psychologists, dietitians, diabetes specialist nurses, mental health nurses and other staff members (MDT co-ordinators, administrators).

Once in post, staff reported a very positive experience of working in the T1DE teams. In the survey, staff agreed with the questions ‘I value the impact the T1DE service has had on my work’ (average score 4.52/5) and ‘I believe that participating in the T1DE service is a legitimate part of my role’ (average score 4.75/5) (online supplemental appendix 2).

During interviews, staff talked about how good an opportunity it was to work in the T1DE service. Some also saw the wider benefits in being able to apply learning to other job roles (many worked part-time in T1DE) and how they had generally become more ‘psychologically minded’.

Many saw the benefit of working within the integrated T1DE team and how they were able to offer better targeted care by treating T1DE as a single entity rather than treating disorders separately.

I’m really seeing how beneficial that can be when you do have it [clinical psychology], not just with disordered eating but how beneficial it could be for some of our other clients struggling with other challenges

However, it was also recognised this was a challenging group to work with and some staff talked of the emotional toll and how patients would keep them awake with worry at night or over weekends.

we in mental health are able to tolerate risk in terms of self-harm and suicide…likewise we had to tolerate high levels of ketones and glucose as part of the treatment initially and that would scare me

This risk was managed as a part of the MDT and staff also had quick and easy access (via eg, email, phone, WhatsApp groups) to other team members. Peer support and clinical supervision were highlighted as being important to help manage stress and provide reassurance. Some staff in existing services talked about the need to retain boundaries by setting ground rules for patients from the beginning.

because we’ve never learned boundaries and there’s a reason for that, because if you’ve got a busy clinic you’re seeing whoever comes through the door, you can’t say okay, I’ve seen my 50 patients today, it’s time to go home, goodbye.

Some staff also talked about how previously they had struggled with the language and ability to cope with this patient group.

learn new language and you learn new words and you learn new concepts and, you know, all the psychological stuff that… you’d no idea about.

One aspect that some staff at existing services recognised as being potentially problematic was the discharge of patients, as there was sometimes a reluctance to discharge, particularly for staff from diabetes teams. It was also recognised that patients found discharge difficult and disliked the word ‘discharge’ and its connotations. Some staff felt it was important to prepare patients well in advance.

[we] hadn’t really clocked that people with diabetes might not realise that this service is an entity that they will come in, have treatment and leave

Staff in existing services talked about the frustration and difficulty of obtaining funding once the original NHS England pilot finished. Uncertainty of funding led to a stop-start service (‘a hiatus in recruitment’) and year-on-year funding was reported as being disruptive and led to increased clinical risk in patients. Ongoing funding was also recognised as important to new T1DE services.

I think we all know that mental health is always the first to go when there’s austerity measures. Anything that looks at wellbeing that isn’t quite as much hard sciencey is what goes. But also there’s this, the technology takeover of diabetes at the moment means that I feel like probably a lot of ICBs think they’re like, well, we can have psychology, or we can have pumps….So my worry is that when this pilot finishes, diabulimia will have had its moment in the sun….what’s going to happen is that diabulimia won’t be sexy anymore. T1DE won’t be interesting anymore.

### Patient experience

Interviews were conducted with nine patients (eight from new T1DE services and one from an existing service—average duration 62 min). Six interviewees came from one T1DE service and one each from three other services.

All interviewees were female and white/white British. Three were under 30 years, three 30–40 years and three over 40 years. Six were diagnosed with type 1 diabetes in childhood or adolescence, and the majority were diagnosed with an eating disorder in the last 5 years.

Patients found that care provided by the T1DE services was very different to previous care. Previous experience of care was reported as being disjointed and based on a medical model with the type 1 diabetes and the eating disorder (and/or mental health) treated separately, or where the eating disorder was largely ignored. Previous care within diabetes clinics was reported as being focused on clinical parameters rather than on how individuals were feeling or coping. Some patients did not recognise they had an eating disorder but had been given the impression that they were a ‘bad’ or ‘poor’ diabetic. This often resulted in individuals feeling isolated and sometimes disengaging from services.

in the past a lot of consultants are only bothered about the numbers and they actually don’t care and I think throughout my first probably ten, 15 years of having diabetes, I don’t think anyone ever asked me, like, how I was or how I felt about any of it. I think it was all, yes literally like, well let’s just tick off these numbers. Oh, they’re all good. Oh yes. Yes, you’re healthy on paper, therefore off you go.

Prior to the T1DE service, patients used terms such as being at a point of ‘burnout’, wanting help to pull themselves out of a ‘black hole’ or how they were at ‘rock bottom’. Some were seeking care to avoid developing later complications.

All patients reported feeling very supported and that staff within the T1DE services had a good understanding and knowledge of T1DE, with the majority reporting they felt listened to in a non-judgemental way. This enabled patients to feel comfortable and safe, allowing them to be more open and honest. They also felt that members of the T1DE teams knew them as individuals and care provided was tailored to their individual needs.

I feel like they understand me more, then it makes me actually, sort of, open up and talk more about what I’m thinking and feeling because the other times, if I’m like, well you don’t know who I am. You don’t care. I’m going to walk out this room and I don’t trust that you’re going to do X, Y and Z that you said you’re going to do.

Patients appreciated the ability to choose options they felt they would benefit from (eg, talking therapy; dietetic advice; diabetes advice) despite services not being fully operational. Services provided sessions at a pace to suit patients (eg, weekly or 2 weekly), generally working in gradual steps and offering practical tips and advice (eg, how to manage conditions while on holiday). Services were reported as being adaptable to needs and flexible in terms of whether appointments were face-to-face or online depending on patient preference. Patients also described how services were quick to respond via email and/or phone call.

Outcomes identified as being important to patients included health, resilience, well-being and quality of life. Some patients also talked about how their physical health could only start to change once there were improvements in mental health. Others felt it was important to gain skills, knowledge and confidence to cope rather than going back to adopting behaviours that might be harmful or self-sabotaging.

I’d love to maintain that ability to put in different coping strategies rather than going to the ones that hurt me

Although such improvements were aligned with the integrated care model underlying T1DE, it was less clear how such changes in patient experience would feature in decisions about commissioning.

### Patient outcomes and associated costs

There were 198 referrals to the five new pilot services, 150 (76%) completed an initial assessment and 139/150 (93%) were admitted to T1DE services. The majority of referrals (n=76, 38%) and those accepted on the care pathway and receiving care (n=51, 37%) came from one service. Three of the remaining services had a similar number of patients accepted on the care pathway (n=24/25). Of those accepted on the care pathway, 89% were female and 92% were white.

6-month follow-up had been completed for 47 patients (18 from one service). For those still in the service, the median time ranged from 6 to 13 months.

Table 2 shows the clinical outcomes from the MDS at baseline and 6 months. The key clinical outcome, HbA1c, showed a slight improvement with a mean reduction of 0.97% (11.2% to 10.2%) at 6 months, which would typically be considered as clinically significant. The DEPS-R scale, which has a clinical threshold of 20 or more to identify patients with T1DE, had a high mean score of 46.1 at baseline, decreasing to 41.2 at 6 months. DDS-2 improved from 4.89 to 4.29 but was still above the clinical threshold for high diabetes distress. At baseline, other health outcomes (GAD7, PHQ9 and WSAS) had higher mean scores than recommended clinical thresholds at baseline. At 6 months there was a small (positive) reduction in all measures; however, all measures remained above the clinical threshold, except for the mean score on the PHQ-9 which improved by 1.3 points (from 16.2 to 14.8) to just below the clinical threshold of 15 (indicative of moderately severe or severe depression)—however, this would not be considered a minimal clinically important difference.^17^

**Table 2:** Health outcomes measures from the minimum dataset at baseline and 6-month follow-up

Consequence	N at both time points	Mean at baseline	Mean at 6-month follow-up	Mean difference from baseline
HbA1c (mmol/mol)	47	98.5	87.9	−10.6
HbA1c (%)	47	11.2	10.2	−0.97
BMI (kg/m^2^)	46	23.9	24.2	0.32
DDS-2	31	**4.89**	**4.29**	−0.60
DEPS-R	32	**46.1**	**41.1**	−5.05
GAD7	32	**13.7**	**12.8**	−0.88
PHQ9	32	**16.2**	14.8	−1.34
EQ-5D-5L	29	0.65	0.66	0.01
WSAS	32	**19.5**	**17.5**	−2.00

Possible scores and clinical thresholds: figures in bold indicate those above clinical thresholds.

DDS-2: scored 1–6, with scores >3 indicating high diabetes distress.

DEPS-R: scored 0–80, clinical threshold 20+ used to identify patients with T1DE.

GAD-7: scored 0–21, clinical threshold 10+ to identify generalised anxiety disorder.

PHQ-9: scored 0–27, clinical threshold 15+ indicative of moderately severe or severe depression.

WSAS: scored 0–40, clinical threshold >10 indicates significant functional impairment.

BMI, body mass index; DDS-2, Diabetes Distress Scale – 2; DEPS-R, Diabetes Eating Problem Scale – Revised; GAD, Generalised Anxiety Disorder; HbA1c, glycated haemoglobin; PHQ, Patient Health Questionnaire; T1DE, type 1 diabetes disordered eating; WSAS, Work and Social Adjustment Scale.

The economic costs for running the T1DE services varied significantly across regions, reflecting differences in population, resource use and service priorities (table 3). Service 4 recorded the highest total expenditure at £415 481 but also had the highest number of patients accepted on the care pathway and therefore the lowest cost per patient of £8 147. Service 5 had a similar total expenditure of £409 776 but with only 24 patients accepted on the care pathway had the highest per patient cost (£17 074).

**Table 3:** Referrals, accepted on care pathway and costs by individual services

	Service name				
	1	2	3	4	5
Referrals	16	45	27	76	34
Accepted on care pathway	14	25	25	51	24
Total cost to end June 2025*	£211 124	£336 299	£278 065	£415 481	£409 776
Per patient cost on care pathway	£15 080	£13 452	£11 123	£8 147	£17 074

* Cost data available up to October 2024 while clinical outcomes are reported up to June 2025. To ensure comparability the average monthly cost was calculated and extrapolated for the remaining 9 months.

The expectation would be that costs would reduce once services become fully embedded and patient throughput increases, which may in turn lead to per patient costs equalising over time.

## Discussion

### Summary

In this evaluation, T1DE models in different locations displayed modest variation, with similarities across services being more apparent. In terms of providing an integrated service, this involved the use of MDTs, joint clinical appointments, peer and clinical supervision. Some services also provided a ‘consultation’ role providing support and advice to wider networks. Implementation was delayed at many services due to challenges recruiting key staff. Once established, staff and patients were very positive about the service.

One aspect identified as a potential problem was the discharge of patients and since patients have longstanding relationships with their local diabetes teams, there is a worry that this could create inherent expectations/tensions with T1DE services. However, staff in services spoke about the importance of working alongside local diabetes services and that discharge from T1DE was likely to take a gradual step-down approach.

Patients reported markedly different care to anything they had received in the past and clinical outcomes showed an improvement in all health measures, particularly for diabetes outcomes, although numbers with follow-up data were relatively small and there was no comparative data, limiting conclusions about clinical or cost effectiveness. Nevertheless, the reduction in HbA1c may be considered as clinically significant,^12 13^ although this must be viewed in the context of other factors including age, gender, ethnicity, comorbidity and baseline HbA1c. Other health-related outcomes, for example, outpatient attendances, inpatient admissions and Accident & Emergency (A&E) attendance are also likely to be important in assessing the clinical and cost-effectiveness of these services. These will be explored further as a part of an NHS England report.

The per patient costs ranged from approximately £8 150 to £17 100 depending on stage of implementation. The costs are, however, likely to reduce once services become fully embedded and patient numbers increase. There was no control (or treatment as usual) group to compare the costs to those accepted onto the T1DE care pathway. The literature is limited on the cost of services which aim to integrate physical and mental health services. A systematic review conducted in 2020 identified 43 studies that integrated physical and mental healthcare for a range of conditions,^23^ of which three studies were conducted within the UK, one of which examined healthcare costs. The study integrated psychological and social care for poorly controlled diabetes using a case management approach.^24^ Psychological interventions included cognitive–behavioural therapy, motivational interviewing and family work, while social interventions included advocacy, support with financial, housing, employment, socialising and literacy. The study estimated a net saving of £12 000 per annum based on reductions in A&E attendance, bed days and non-attendance rates. At the time of the current evaluation, these outcomes were not available but will be reported at a later date, in an NHS England report.

### Strengths and limitations

A strength of the current study includes the mixed methodology approach gathering views from a range of data sources including qualitative interviews with a large number of staff with a range of job roles from new and existing T1DE services; survey data; and detailed economic costing data.

A limitation of the current study was that implementation and set-up of services was much slower than anticipated, and as a consequence, the number of patients recruited to T1DE services was low. This had implications in terms of health outcome data and economic analysis, but also in terms of those that could be invited for interview. Patient interviews were conducted primarily from one service, making it difficult to draw conclusions about how patient experience may vary depending on how services were configured (although patients at each of three other services appeared to report similar experiences). In addition, all patients who took part in the qualitative interviews were female and white/white British and therefore may not represent the wider T1DE population. T1DE is reported as being more prevalent in females,^25 26^ but one study has suggested that insulin restriction or omission may be just as prevalent among males.^27^ The evidence in relation to ethnic origin is lacking, however, some ethnic minorities^28 29^ have higher levels of HbA1c and increased incidence of hypoglycaemia, which may be associated with the risk of T1DE. Ethnic minorities may also face additional barriers to diagnosis and treatment of T1DE.^29^ Patients were approached by staff at local T1DE services to invite them to take part in interviews. Staff may not have approached those they deemed as unsuitable for interview. Finally, it was not possible to incorporate a comparison or control group; therefore, we could not demonstrate changes in relation to those not receiving the T1DE service.

### Comparison with previous literature

Little has been published on integrated services for T1DE. Available literature (published and unpublished) comes from the evaluation of the two earlier pilot T1DE services previously commissioned by NHS England.^630^,^32^ Delivery models in the previous evaluation worked in similar ways to those in the current evaluation. Newer pilots learnt from existing services (eg, by sitting in on joint clinic appointments and/or MDT meetings), and, through an annual conference. The most important facilitators identified in the previous evaluation were joint (integrated) working, MDT meetings and access to expert advice and support. However, similar issues were also reported in the previous evaluation in terms of workforce issues including staffing, backfill of posts and working across different information technology systems, in addition to space and ongoing commissioning. By the end of the current evaluation, two of the three original pilots had closed in their present form due to challenges in securing local funding from their Integrated Care Board (ICB) following the initial period of investment. Ongoing commissioning was identified as a major barrier to sustainability of the T1DE services—particularly for existing services, despite a move in the right direction in terms of health outcomes.

Overall, 73% of patients in the previous evaluation reported being satisfied with the treatment received and recognised the importance of integrated care (diabetes and mental healthcare) received from the T1DE services and the skills and knowledge this brought. They also reported improved outcomes in terms of health and well-being. This concurs with the findings from the current evaluation.

Compared with the previous evaluation,^6^ there was a smaller reduction in HbA1c (0.97%) compared with reductions in the two earlier pilots (2.3% and 2.5%). This may be due to the small numbers (in both evaluations) and the shorter length of follow-up in the current evaluation (6 months compared with 12 months in the previous evaluation). More recently published data for the London T1DE service^32^ on 47 patients with 12-month follow-up showed a statistically significant reduction in HbA1c (0.92%, 95% CI 0.31 to 1.54, p=0.0047). Reductions were also seen in DEPS-R, PHQ-9 and GAD-7, the latter demonstrating a statistically significant reduction in anxiety.

### Implications for future research

While the staff and patient experience of T1DE services (new and existing services) has been largely positive and much has been learnt about delivering care to patients via integration of physical and mental health services, the evidence base for the effectiveness and cost-effectiveness of T1DE services remains limited despite the implementation of the current and previous pilot study.

Pilots are necessary to provide evidence for later commissioning but face a paradox—lack of evidence for a new model means that investment is necessarily time limited and bounded, which in turn means that insufficient time may be available to accrue enough evidence to demonstrate the sort of significant, long-term effects that would support expanded commissioning of the service.^33^,^35^ The current evaluation highlights that the set-up of pilot services takes time and is intensive. Several challenges were encountered by all services, including recruitment of key staff. Services require time to gain knowledge, understanding and confidence to work with this high-risk group and facilitating the need to build trust and relationships with patients. Pilots for low volume, high-cost services are particularly risky and while starting small makes sense, sufficient time must be given to accrue health outcomes in order to generate evidence of service impact. Furthermore, in order to ensure services are sustainable and commissioned at local level, consideration may need to be given to alternative commissioning footprints and service delivery models.

Due to the high level of risk, services focused on gradual changes in patient behaviour, therefore outcomes, as measured in the MDS, may take longer to observe than in other patient groups. In addition, longer-term benefits were not captured by the data available to the evaluation. These may include the prevention of future health complications via better diabetes management, which in turn may lead to reduced healthcare costs in the longer term. As more data becomes available over time, outcomes related to additional healthcare resources, such as inpatient stays, hospital admissions for DKA, A&E admissions and other outcomes from primary and secondary care datasets should be investigated.

Other benefits extend beyond those reported in the MDS and future health outcomes. For example, staff talked about approaching all consultations incorporating both physical and mental health using the skills and knowledge they had developed as a part of the pilot. Some services also provided training sessions to other networks and services to help to raise awareness of T1DE and T1DE services (eg, to healthcare professionals in primary care—general practitioners and practice nurses, as well as staff in A&E departments).

### Implications for service delivery

Uncertainty around funding made it difficult to maintain a stable service, with some delaying or halting patient recruitment until additional funding was in place. While it was outlined in the NHS England guidance for expressions of interest that services would be expected to commit to developing plans for a sustained service, further support around developing business cases may have been useful. The T1DE service is a low-volume, high-cost service involving a high-risk group of patients making it challenging to integrate into a ‘business as usual’ model at Trust and ICB level in its current form (two of the existing pilot services closed during the current evaluation).

Consideration may need to be given to the minimum in terms of staff and infrastructure necessary to deliver T1DE services and to what sort of model of delivery would work best. For example, a specialised service model, whereby services are planned nationally and delivered regionally by experts with the necessary skills and experience, may be an option, offering different care pathways depending on patient need, including a greater focus on providing local teams with advice and support through a ‘consultation’ approach. Such a service might be modelled along the lines of the maternity services model for women at high risk of complications during pregnancy (https://east-midlands-maternal-medicine-network.nhs.uk/). This model offers a number of care options depending on service user need, including advice regarding pregnancy care, shared care with local teams or care delivered by maternal maternity centres. Specialist topic MDTs may also be attended by all clinicians caring for pregnant women.

The challenge will be to replicate the aspects of the current T1DE service that are most favourable (such as positive patient experience, as well as confidence and capacity building among staff) using a model which is more focused on advice and support, rather than direct service provision, and is delivered remotely.

Given the lack of knowledge and confidence in identifying T1DE among healthcare professionals,^36 37^ consideration should also be given as to how best to retain and use staff recruited to pilot services as they have gained invaluable knowledge, understanding, experience and skills working with this specific group of patients.

## Supplementary material

10.1136/bmjopen-2025-107381 online supplemental file 1

10.1136/bmjopen-2025-107381 online supplemental file 2

10.1136/bmjopen-2025-107381 online supplemental file 3

10.1136/bmjopen-2025-107381 online supplemental file 4

## Data Availability

No data are available.
