# Supplementary material for: Delivering integrated diabetes and mental healthcare for people with type 1 diabetes disordered eating (T1DE): a mixed methods evaluation
Source: BMJ Open. 2026 Mar 9;16(3):e107381. doi: 10.1136/bmjopen-2025-107381 (PMC12983690; doi:10.1136/bmjopen-2025-107381)
Supplement: online supplemental file 1 [file bmjopen-16-3-s001.docx]

**Appendix 1: Staff topic guide**

**Topic guide: Type 1 diabetes disordered eating (T1DE) services**

**General introduction and background**

1. Could you describe your role within the T1DE integrated service?
   1. What is your background?
   2. What specific training, if any, have you received related to the T1DE integrated service? – prompt: formal training, shadowing, MDT meetings etc
      1. What are your experiences with training provided as part of the integrated service?
      2. Was sufficient training provided, if not why not and what additional training would you have liked?
      3. Learning from other sites – why?
      4. Were people with lived experience of T1DE able to contribute to the training?
2. What are your areas of responsibility related to the integrated T1DE service?
   1. How do these relate to your previous responsibilities? - prompt: similarities, differences, changes

**T1DE service integration and implementation**

1. What does the integrated model look like in your area?
   1. What staff are in the service? What staff have been newly recruited and why? Were service users involved in the interview process/panel?
   2. Where does the service sit? Physical location – acute or MH Trust? Diabetes or ED services? Why?
   3. Existing service.
   4. Existing liaisons and relationships among healthcare providers and others.
   5. How are the T1 Diabetes services and Disordered Eating services integrated?
      1. Prompt: Communication between staff in service, integration of treatment.
      2. Prompt consistency of treatment delivery.
      3. Prompt: Transitions into and out of service.
      4. Multi-disciplinary team (MDT) meetings – how does it work?
2. What was the rationale behind the T1DE integrated model in your area?

*Here, please specify local contextual information relevant to T1DE provision and patient need*

1. How was the integrated service developed?
   1. Who provided input into service development and how 🡪 patients, voluntary sector, ICS etc.
   2. How does the developed service relate to the NHS-England specification?
      1. How does it differ from NHS England specification and why?
      2. How has it changed over time? Why?
2. What are expected barriers to implementation of the integrated service/What barriers have you experienced during implementation of the integrated service?
3. What are expected barriers to running the integrated service/What barriers have you experienced in running the integrated service?
4. What are expected barriers to continuation of the integrated service after funding for the pilot runs out?/What barriers to continuation of the service have you experienced after the pilot funding ran out? What parts of the infrastructure are necessary to maintain the service?
5. What would facilitate implementation of the integrated services/what facilitated implementation of the integrated service?
6. What would facilitate running of the integrated service/what facilitated running of the integrated service?
7. What would facilitate continuation of the integrated service after pilot funding runs out?/What facilitated continuation of the integrated service after pilot funding ran out? Have services proactively sought advice on future joint working/funding streams from the voluntary sector?
8. What are your views on further commissioning of the service? How might this work?

**Experience and acceptability**

1. What are your experiences with care delivery within the integrated service?
   1. What are your experiences of treating this patient group pre-T1DE service.
   2. How has the T1DE service impacted on this?
2. What is going well?
3. What is going less well?

1. Acceptability – how is it working for you?
   1. Risks and responsibilities – prompts re. emotional toll? better or worse
   2. how does it impact on how they enjoy their job/ stress taken home etc?
2. How do you think other staff view T1DE (acceptability) and their experience of these, including how staff interface with other clinical teams?
3. Do you have regular clinical supervision meetings? How often? Who with? Have these been helpful?
4. Are there escalation/ de-escalation protocols in place – have you had to use them?
5. What impact do you think T1DE has had on service users?
   1. Which outcomes have you used to measure impact on service users?
   2. What do you feel are the important elements of the integrated care service that impact on patient outcomes?
   3. What outcomes do you think are important to service users?
   4. What outcomes do you consider show that the pilot has been successful – reduced HbA1c, increased ‘time in range’, better depression score, patient weight, patient feedback (slightly different focus to 18a as this focuses on organisational priorities).

**Patient / care pathway**

1. Who is referred /criteria for referral? Who is admitted to the service? Catchment area – wider area?
   1. How many patients can the service see at any one time – capacity?
   2. Is any support provided to those not eligible for the service?
   3. What happens to those that DNA / fail to engage (are there policies in place?)
   4. What happens to those who refuse treatment (is there a policy in place?)
   5. Is there an equity health impact assessment?
   6. For those referred to the service, how is location of the diabetes care team agreed, what does this depend on?
   7. Approaches from under 18s, how is this dealt with?
2. What support and treatment is provided as part of the T1DE service?
   1. How do you decide what treatments to offer? Prompt -tailor to patient need - patient centred?
   2. What other support does the T1DE service offer?
   3. How do you keep service users engaged?
   4. What happens if patients need to be hospitalised during their treatment in T1DE?
   5. What are local eating disorder services criteria for admission?
   6. How well established are these links? Which provider? What are the relationships like? Communication, co-ordination, consistency in approach – how is this maintained?
   7. Has the T1DE team had to section anyone? How did that go?
3. How many service users have been discharged? How long have they been in the service on average – time limited treatment? What are the reasons for discharge? How many have been referred back into the service?
4. When patients are discharged from the integrated service what support/ follow-up is available to them? How do service users view the discharge process?
   1. How is transition to other services organised?
   2. How is relapse dealt with?
5. Are there any care pathway links with other services? Prompts - primary care, including GPs and practice nurses, local emergency departments, acute medical units, local mental health liaison psychiatry and eating disorder services, social and pastoral care, higher and further education providers and community sector.
6. Please can you describe these links?
7. Links with charitable sector e.g. BEAT, JDRF?
8. What is the balance between direct service delivery and consultation? Barriers to consultation (upskilling?)
9. What do you think are potential, or wider effects, (favourable and unfavourable) of the integrated service, if any? For example: learning effects – improvement in skills/knowledge/ awareness; shared resources; other benefits/disadvantages to non-T1DE patients
   1. Educational outreach?
   2. Educating family members and carers
