# Supplementary material for: Delivering integrated diabetes and mental healthcare for people with type 1 diabetes disordered eating (T1DE): a mixed methods evaluation
Source: BMJ Open. 2026 Mar 9;16(3):e107381. doi: 10.1136/bmjopen-2025-107381 (PMC12983690; doi:10.1136/bmjopen-2025-107381)
Supplement: online supplemental file 2 [file bmjopen-16-3-s002.docx]

## Appendix 2: Staff survey

The staff survey included the validated Normalisation MeAsure Development questionnaire (NoMAD) (Finch et al. 2015) and the Acceptability of Intervention Measure (AIM), Intervention Appropriateness Measure (IAM), and Feasibility of Intervention Measure (FIM) (Weiner et al. 2017)

NoMAD was designed to measure constructs of Normalisation Process Theory (NPT) exploring factors that support or inhibit embedding of new practices into normal care (May et al. 2009). NPT constructs include:

- coherence (sense-making individually and collectively);
- cognitive participation (legitimisation of practice and commitment by participants);
- collective action (relational and contextual integration including skills, training and resources);
- reflexive monitoring (comprehension of the effects of practice and appraisal by participants)

Three general questions on the NoMAD questionnaire were scored on a Visual Analogue scale from 0 to 10 where 0 = no agreement and 10 = complete agreement with each statement.

All other measures (remaining NoMAD questions, AIM, IAM, FIM) were scored on a Likert scale from strongly/ completely agree to strongly/ completely disagree for each statement and scored on a scale of 1-5 (where 5 represented strong agreement). Scores were averaged across respondents.

## Results from the staff survey

## Table A1: Mean scores on the NoMad questionnaire - general questions about the service (rated on a scale from 0-10)

|  | **All services** | **New pilots** | |
| --- | --- | --- | --- |
|  | **Baseline (n=26)** | **Baseline (n=20)** | **Follow-up (n=20)** |
|  | Mean (SD) | Mean (SD) | Mean (SD) |
| How familiar does the T1DE service feel? | 5.70 (2.98) | 5.05 (2.86) | 6.80 (1.91) |
| T1DE service currently a normal part of work? | 5.74 (3.50) | 6.05 (3.20) | 7.20 (2.31) |
| T1DE service will become a normal part of work? | 6.93 (3.08) | 7.40 (2.74) | 6.70 (3.11) |

SD = standard deviation

## Table A2: Mean scores on the NoMAD questionnaire at baseline for all staff (n=26) and staff in new pilot services (n =20) and for staff at new pilot services at follow-up (n=20) (rated on a Likert scale from 1 to 5)

|  | **All services** | **New pilots** | |
| --- | --- | --- | --- |
|  | **Mean score baseline** | **Mean score baseline (n=20)** | **Mean score follow-up (n=20)** |
| **Coherence** |  |  |  |
| I can see how the T1DE service differs from usual ways of working | 4.44 | 4.37 | 4.32 |
| Staff in this organisation have a shared understanding of the purpose of the T1DE service | 4.04 | 4.05 | 4.00 |
| I understand how the T1DE service affects the nature of my work | 4.22 | 4.12 | 4.16 |
| I can see the potential value of the T1DE service | 4.81 | 4.75 | 4.65 |
| **Cognitive participation** |  |  |  |
| There are key people who drive the T1DE service forward and get others involved | 4.54 | 4.53 | 4.30 |
| I believe that participating in the T1DE service is a legitimate part of my role | 4.75 | 4.83 | 4.32 |
| I’m open to working with colleagues in new ways to deliver the T1DE service | 4.88 | 4.83 | 4.53 |
| I will continue to support the T1DE service | 4.65 | 4.70 | 4.58 |
| **Collective Action** |  |  |  |
| I can easily integrate the T1DE service into my existing work | 3.57 | 3.88 | 3.83 |
| The T1DE service disrupts working relationships* | 4.08 | 4.05 | 4.32 |
| I have confidence in other people’s ability to deliver the T1DE service | 4.19 | 4.50 | 4.10 |
| Work is assigned to those with skills appropriate to the T1DE service | 4.08 | 4.21 | 4.25 |
| Sufficient training is provided to enable staff to implement the T1DE service | 3.69 | 3.80 | 3.80 |
| Sufficient resources are available to support the T1DE service | 3.08 | 3.60 | 3.65 |
| Management adequately supports the T1DE service | 3.69 | 4.10 | 3.75 |
| **Reflexive monitoring** |  |  |  |
| I am aware of reports about the impact of the T1DE service | 4.20 | 4.05 | 3.65 |
| The staff agree that the T1DE service is worthwhile | 4.60 | 4.58 | 4.45 |
| I value the impact that the T1DE service has had on my work | 4.52 | 4.47 | 4.42 |
| Feedback about the T1DE service can be used to improve it in the future | 4.68 | 4.68 | 4.55 |
| I can modify how I work with the T1DE service | 4.50 | 4.44 | 3.83 |

Scores range from 1-5, where 1 = strongly disagreed to 5 = strongly agreed; higher scores represent higher agreement with a particular statement. *Scores on the statement ‘The T1DE service disrupts working relationships’ were reverse coded.

## Table A3: Mean scores on the Acceptability of Intervention Measure (AIM), Intervention Appropriateness Measure (IAM), and Feasibility of Intervention Measure (FIM) (rated on a Likert scale from 1 to 5)

|  | **All services** | **New pilots** | |
| --- | --- | --- | --- |
|  | **Mean score baseline (n=26)** | **Mean score baseline (n=20)** | **Mean score follow-up (n=20)** |
| **Acceptability of T1DE** |  |  |  |
| The T1DE service meets with my approval | 4.15 | 4.05 | 4.20 |
| The T1DE service is appealing to me | 4.38 | 4.35 | 4.40 |
| I like the T1DE service | 4.35 | 4.30 | 4.50 |
| I welcome the T1DE service | 4.54 | 4.50 | 4.65 |
|  |  |  |  |
| **Appropriateness of T1DE** |  |  |  |
| The T1DE service seems fitting | 4.42 | 4.40 | 4.40 |
| The T1DE service seems suitable | 4.42 | 4.35 | 4.40 |
| The T1DE service seems applicable | 4.46 | 4.40 | 4.45 |
| The T1DE service seems like a good match | 4.46 | 4.35 | 4.55 |
|  |  |  |  |
| **Feasibility of T1DE** |  |  |  |
| The T1DE service seems implementable | 4.27 | 4.35 | 4.10 |
| The T1DE service seems possible | 4.38 | 4.50 | 4.05 |
| The T1DE service seems doable | 4.42 | 4.50 | 3.90 |
| The T1DE service seems easy to use | 3.92 | 3.95 | 4.05 |

Scores range from 1-5, where 1 = completely disagree to 5 = completely agree; higher scores represent higher agreement with a particular statement.

## References

1. Finch TL, Girling M, May CR et al. (2015). NoMad: Implementation measure based on Normalization Process Theory. [Measurement instrument]. Retrieved from http://www.normalizationprocess.org.

2. Weiner, B.J., Lewis, C.C., Stanick, C. et al. Psychometric assessment of three newly developed implementation outcome measures. Implementation Sci 12, 108 (2017). <https://doi.org/10.1186/s13012-017-0635-3>

3. May C, Mair FS, Finch T et al. Development of a theory of implementation and integration: normalization process Theory. Implement Sci. 2009;4:29.
