# Supplementary material for: Delivering integrated diabetes and mental healthcare for people with type 1 diabetes disordered eating (T1DE): a mixed methods evaluation
Source: BMJ Open. 2026 Mar 9;16(3):e107381. doi: 10.1136/bmjopen-2025-107381 (PMC12983690; doi:10.1136/bmjopen-2025-107381)
Supplement: online supplemental file 3 [file bmjopen-16-3-s003.docx]

**Service user experiences of Type 1 diabetes disordered eating (T1DE) services**

**Topic guide**

We would like to get your insights into the receipt of care from the Type 1 diabetes disordered eating service (T1DE) at xxxxxx. **You do not have to discuss anything you do not wish to.**

**Background**

- How long have you received care in the integrated T1DE service? And how long do you anticipate being in the service?
- What treatment did you receive prior to the T1DE service? Where did you receive this treatment?
- What was your experience like prior to T1DE service?

**Awareness/ knowledge/ access of T1DE services**

- How did you first learn out about the integrated T1DE service?
- What did you think about posters/flyers/social media about the service?
- What did you expect of the T1DE service? What were you told about the service and how it worked? e.g. number of sessions (time limited)?
- Who referred you to the service and why?
- What has been your experience of referral to the T1DE service?
  - Prompt – communication, waiting list
- What has been your experience of accessing the T1DE service?
  - Prompt – how easy? affordability of accessing services, resources available in service meet needs of service user, geographic accessibility of service, accommodation of service to meet constraints and preferences of patient, acceptability of service
- How flexible is the T1DE service?
  - Prompt – changing appointments, attendance.

**Care in the T1DE service**

- Experience of care in the T1DE service
- What treatment / advice have you had as a part of the T1DE service?
- Compared to the care you received prior to accessing the T1DE service, has the care you receive changed, and if so, in what way? How does it differ? What additional treatment/ therapies have you had access to?
- Has the integrated T1DE service been helpful to you, and if so, in what way? Or why not? How has it impacted on you? Prompt: skills, coping mechanisms, education & tools

**Integration of care in the T1DE service**

- What do you think of the integration of T1 and DE services? How has that worked? What do you think of staff knowledge/ training? Have you felt understood?

Prompts - consistent/contradictory advice? telling your story/history multiple times? does the service feel joined up and working for you? co-ordination of own care?

Co-ordination of care/ joined up approach – how has that worked? Organisation/structure?

- Who has been in charge of your care in the T1DE service? How have the services worked together – who has led that? Have there been any changes in staff and how has this impacted your treatment?
- How does the service link to your local team/s -communication
- Do you feel supported? In what way? If not, why not?

**Outcomes in the T1DE service**

- What outcomes are important to you?
  - Prompt – health (hydration), DKA admissions/ hospitalisations (prior, during, after), social, work, sleep (quality), energy, motivation etc; do you feel these outcomes can be achieved
  - What are your thoughts about the questionnaires you have been asked to complete?
- How is your relationship with T1 and ED now? How has it changed since being in the T1DE service?

**T1DE Service advantages/ disadvantages/ expectations**

- What elements of the T1DE service do you think worked well, and why? And what elements have not worked so well and why not?
- What do you think are the benefits/ advantages of the T1DE service? And disadvantages?
- Has the T1DE service met your expectations? Is there any way the T1DE service could be improved?
- Would you recommend the T1DE service to someone else if they needed it?
- What do you think are the important key elements of the service? What would you change/add/remove from the service?

**Discharge (for those discharged)**

- Experience on discharge from the service & longer term in terms of disordered eating & diabetes
- How long were you in the service for?
- How did you feel on discharge from the service?
- What did discharge of the service look like? What kind of continued support is provided, if any? Relapse prevention plans/ strategies?
- What skills /coping mechanisms/ tools/strategies have you gained to help you moving forward?
- Were you referred to other services e.g. GP, ED, social care, mental health? Was support/education/ knowledge provided to others around you e.g. family members/ carers
- How has handover to your primary point of care clinician (if different from T1DE service) been? Is there increased awareness of T1DE? Is it any different from previous care? Strategies/ support/ guidance for local teams?
- How much communication between local diabetes & T1DE service – discharge meeting, key contact at T1DE service, teaching local teams? How has that been managed – patient led, adhoc, protocol?

**Other**

- Unintended consequences of T1DE service – increased awareness other health care professionals, family members
