# Supplementary material for: Delivering integrated diabetes and mental healthcare for people with type 1 diabetes disordered eating (T1DE): a mixed methods evaluation
Source: BMJ Open. 2026 Mar 9;16(3):e107381. doi: 10.1136/bmjopen-2025-107381 (PMC12983690; doi:10.1136/bmjopen-2025-107381)
Supplement: online supplemental file 4 [file bmjopen-16-3-s004.docx]

## Appendix 4: Economic costs

We conducted a cost-consequences analysis to explore service resource use and associated cost data alongside interim changes in health outcomes to give insights into care costs versus health consequences. The REVAL team undertook an economic analysis using the anonymised aggregated clinical data from the MDS supplied by NHS England analysts and associated costing information.

To collect information on staff input and other healthcare resources, pay and non-pay costs, a detailed questionnaire (see below) was distributed to key contacts in each service. For staff pay costs details were collected on job role expressed in terms of % full-time equivalent (FTE) and grade. Capital costs were excluded from the analysis.
