# Supplementary material for: Delivering integrated diabetes and mental healthcare for people with type 1 diabetes disordered eating (T1DE): a mixed methods evaluation
Source: BMJ Open. 2026 Mar 9;16(3):e107381. doi: 10.1136/bmjopen-2025-107381 (PMC12983690; doi:10.1136/bmjopen-2025-107381)
Supplement: Supplementary file 5 [file bmjopen-2025-107381.reviewer_comments.pdf]

PEER REVIEW HISTORY

BMJ Open publishes all reviews undertaken for accepted manuscripts. Reviewers are asked to complete a checklist review form and are provided with free text boxes to elaborate on their assessment. These free text comments are reproduced below.

ARTICLE DETAILS

Title (Provisional)

Delivering integrated diabetes and mental health care for people with Type 1 diabetes disordered eating (T1DE): a mixed methods evaluation

Authors

Harkness, Elaine F.; Arif, Saima; Gillibrand, Stephanie; Wilson, Paul; Dumville, Jo; Bower, Peter; Kletter, Maartje

---

VERSION 1 - REVIEW

**Reviewer**	**1**
**Name**	**Igudesman, Daria**
**Affiliation**	**AdventHealth**
**Date**	**29-Jul-2025**
**COI**	**None**

Thank you for the opportunity to review the manuscript entitled, "Delivering integrated diabetes and mental health care for people with Type 1 diabetes disordered eating (T1DE): a mixed methods evaluation."

Although an important topic, important methodological details are missing that are critical to enable interpretation and reproducibility of this work. The objectives of the study, associated methods, and results do not link together. Critically, the analytic approach is unclear. A definition of health economic analysis is needed, as well as the related outcomes. The abstract alludes to health outcomes, which are missing from the methods of the main body of the manuscript. The results appear to reveal that HbA1c is an outcome, which is not mentioned in methods.

As with the main body of the manuscript, the abstract should clearly link the objective to methods and results, which are at present disconnected. Numeric results are needed in the abstract to match the methods presented (e.g., health outcomes). It is unclear from the abstract what the specific objectives of the analysis were. Is the aim to compare across different services? It is unclear what is mean by "variation." It is unclear why the locations of the health services are mentioned, without any description of the services themselves. The

abstract does not mention timeframe, whereas main body mentions a 6-month study design.

The text should be thoroughly reviewed for grammatical errors and readability (e.g., the title is missing an "and"; first use of "type 1 diabetes" simply states "type 1"). The introduction should have a logical flow of information that leads to the study objectives; it is currently disjointed.

**Reviewer**	**2**
**Name**	**Jones, Christina**
**Affiliation**	**University of Surrey, School of Psychology**
**Date**	**02-Sep-2025**
**COI**	**None**

This is an interesting and highly topical area, given the increasing attention given to T1DE. The focus on implementation of T1DE services from from the perspective of staff and patients is clinically valuable. I have a few areas which would be useful for the team to respond to:

- Whilst the authors have specified that the Normalisation MeAsure Development (NoMAD) questionnaire was utilised, the only mention of the underpinning theory of the questionnaire (Normalisation Process Theory; NPT) is in the appendices which should be expanded on in the methods since readers may not be familiar with implementation theory

- Further information should be provided on how the topic guides were developed with either the topic guides themselves included as supplementary materials or specific questions included as examples rather than broad areas explored

- Should the data presented in Table 1 but aggregated in some way to avoid the services from being identifiable by their team composition (assuming services have been given numeric references to maintain anonymity)

- For some of the results, e.g., the quote around boundaries on p11, it seems that this is more about being seconded from one role into another. Was this something unique to T1DE services which can be expanded on or is there a better quote to support interpretation?

- I also think the finding around discharge is important, since people tend to have long relationships with their T1Ds HCPs this could create inherent expectations/tensions with T1DE services. Could the authors expand on this?

- In terms of the outcomes data, it would be helpful for the authors to reflect on what the minimal clinically important difference is to provide context (e.g., is 0.5% and 5.5 point reduction in HbA1C and DEPS-R clinically meaningful?). Whilst the discussion compares this

with existing published data for T1DE services, MCIDs should be stated where possible for all outcomes

- The specific results related to the health economic analysis should be included briefly rather than deferred entirely to appendix 4. The discussion should expand on the per patient costs in relation to other psychological services/T1D services where possible for context

- There needs to be greater reflection in limitations of the lack of representative findings (all patients were female and White/ White British). How does this compare with what we know about T1DE more broadly?

- Interested to see that the authors have ticked N/A to the need for research characteristics and reflexivity on the SRQR checklist and i'm keen to understand their rationale for not including this information

---

VERSION 1 - AUTHOR RESPONSE

Reviewer: 1
Dr. Daria Igudesman, AdventHealth
Comments to the Author:
Thank you for the opportunity to review the manuscript entitled, "Delivering integrated diabetes and mental health care for people with Type 1 diabetes disordered eating (T1DE): a mixed methods evaluation."

Although an important topic, important methodological details are missing that are critical to enable interpretation and reproducibility of this work. The objectives of the study, associated methods, and results do not link together. Critically, the analytic approach is unclear. A definition of health economic analysis is needed, as well as the related outcomes. The abstract alludes to health outcomes, which are missing from the methods of the main body of the manuscript. The results appear to reveal that HbA1c is an outcome, which is not mentioned in methods.

We thank the reviewer for their comments. We have edited the manuscript to try and make the objectives; methods and results link together better by giving the same headings in each section namely: T1DE delivery models; staff experiences; patient experiences; and health outcomes and associated costs.

We have included a definition of the health economic analysis (pages 8) and health outcomes (pages 7/8). We have added the health outcomes table to the main results (rather than an appendix) on page 15.

As with the main body of the manuscript, the abstract should clearly link the objective

to methods and results, which are at present disconnected. Numeric results are needed in the abstract to match the methods presented (e.g., health outcomes). It is unclear from the abstract what the specific objectives of the analysis were. Is the aim to compare across different services? It is unclear what is mean by "variation." It is unclear why the locations of the health services are mentioned, without any description of the services themselves. The abstract does not mention timeframe, whereas main body mentions a 6-month study design.

Thank you, as noted above we have added consistent headings throughout the paper to help link throughout and to help clarify the objectives. We have added HbA1c results to the abstract – however, the number completing outcomes at 6-month was small. We were interested how implementation differed across services in terms of the models of T1DE services delivered and also in the health economic costs. We have deleted the service names and added to the abstract sthat outcome data was collected at baseline and 6-months.

The text should be thoroughly reviewed for grammatical errors and readability (e.g., the title is missing an "and"; first use of "type 1 diabetes" simply states "type 1"). The introduction should have a logical flow of information that leads to the study objectives; it is currently disjointed.

We refer to T1DE as 'Type 1 diabetes disordered eating' and therefore have not added 'and' to the title. We have modified the first sentence in the introduction. We have reorganised the introduction to try and help improve the flow.

Reviewer: 2
Dr. Christina Jones, University of Surrey
Comments to the Author:
This is an interesting and highly topical area, given the increasing attention given to T1DE. The focus on implementation of T1DE services from from the perspective of staff and patients is clinically valuable. I have a few areas which would be useful for the team to respond to:

We thank the reviewer for their comments.

- Whilst the authors have specified that the Normalisation MeAsure Development (NoMAD) questionnaire was utilised, the only mention of the underpinning theory of the questionnaire (Normalisation Process Theory; NPT) is in the appendices which should be expanded on in the methods since readers may not be familiar with implementation theory

We have expanded on the NoMAD measure in the methods section on pages 6/7.

- Further information should be provided on how the topic guides were developed with either the topic guides themselves included as supplementary materials or specific questions included as examples rather than broad areas explored

Thank you we have added how topic guides were developed (on pages 6 for the staff experience and page 7 for the patient experience). We have also added the topic guides as supplementary material (Appendix 1 & 3)

- Should the data presented in Table 1 but aggregated in some way to avoid the services from being identifiable by their team composition (assuming services have been given numeric references to maintain anonymity)

Thank you for raising this – we have aggregated the data in Table 1 under diabetes, mental health, eating disorders and other. We have also removed the names of the services from the manuscript.

- For some of the results, e.g., the quote around boundaries on p11, it seems that this is more about being seconded from one role into another. Was this something unique to T1DE services which can be expanded on or is there a better quote to support interpretation?

We have added a different quote around boundaries on page 12 which we hope better illustrates the point we were trying to make.

- I also think the finding around discharge is important, since people tend to have long relationships with their T1Ds HCPs this could create inherent expectations/tensions with T1DE services. Could the authors expand on this?

Thank you for comment, we agree that the finding around discharge is important – we have expanded on this in the discussion section on page 16.

- In terms of the outcomes data, it would be helpful for the authors to reflect on what the minimal clinically important difference is to provide context (e.g., is 0.5% and 5.5 point reduction in HbA1C and DEPS-R clinically meaningful?). Whilst the discussion compares this with existing published data for T1DE services, MCIDs should be stated where possible for all outcomes

We have added MCIDs to the methods on pages 7&8 and to the results section on page 14 where appropriate. We have also added to the discussion that the reduction in HBA1c may be considered as clinically significant but must be viewed in the context of other factors (page 16). We moved the table with outcome data from the Appendix to the main body of the paper (page 15) and provide the clinical thresholds and minimal clinically important difference in the methods where available.

- The specific results related to the health economic analysis should be included briefly rather than deferred entirely to appendix 4. The discussion should expand on the per patient costs in relation to other psychological services/T1D services where possible for context.

Thank you for your comment we have included a brief table and summary of the health economic analysis to the results section (page 15). We have also expanded on this in the discussion on page 16.

- There needs to be greater reflection in limitations of the lack of representative findings (all patients were female and White/ White British). How does this compare with what we know about T1DE more broadly?

We have reflected on this on page 17 – thank you.

- Interested to see that the authors have ticked N/A to the need for research characteristics and reflexivity on the SRQR checklist and i'm keen to understand their rationale for not including this information

Thank you for highlighting this, we have added a positionality statement to the manuscript on page 9.

VERSION 2 - REVIEW

**Reviewer**	**2**
**Name**	**Jones, Christina**
**Affiliation**	**University of Surrey, School of Psychology**
**Date**	**29-Oct-2025**
**COI**	

Thank you for adequately addressing the queries raised
